# Novel and Extremely Sensitive NiAl_2_O_4_-NiO Nanostructures on an ITO Sensing Electrode for Enhanced Detection of Ascorbic Acid

**DOI:** 10.3390/molecules29122837

**Published:** 2024-06-14

**Authors:** Asma Hammami, Afrah Bardaoui, Shimaa Eissa, Walid A. M. Elgaher, Radhouane Chtourou, Olfa Messaoud

**Affiliations:** 1 Laboratoire de Chimie, Ecole Supérieure des Sciences et Techniques de la Santé de Tunis, Université de Tunis El Manar, Tunis 1068, Tunisia; 2 U.R Traitement et Dessalement des Eaux, Département de Chimie, Faculté des Sciences de Tunis, 2092 Manar II, Tunisie, Université de Tunis El Manar, Tunis 1068, Tunisia; 3 Laboratory of Nanomaterials and Systems for Renewable Energies (LaNSER), Research and Technology Center of Energy (CRTEn), Techno-Park Borj Cedria, Bp 95, Hammam-Lif, Tunis 2050, Tunisiaradhouane.chtourou@gmail.com (R.C.); 4 Department of Chemistry, Khalifa University of Science and Technology, Abu Dhabi P.O. Box 127788, United Arab Emirates; shimaa.eissa@ku.ac.ae; 5 Center for Catalysis and Separations, Khalifa University of Science and Technology, Abu Dhabi P.O. Box 127788, United Arab Emirates; 6 Helmholtz Institute for Pharmaceutical Research Saarland (HIPS)—Helmholtz Centre for Infection Research (HZI), Saarland University, Campus E8.1, 66123 Saarbrücken, Germany; walid.mohammad@helmholtz-hips.de; 7 Biomedical Genomics and Oncogenetics Laboratory, Institut Pasteur de Tunis, University Tunis El-Manar, Tunis 1068, Tunisia; olfa.messaoud@pasteur.utm.tn

**Keywords:** NiAl_2_O_4_-NiO nanostructures, electrochemical detection, ascorbic acid, electron transfer, catalytic effect

## Abstract

The current study focused on the design of an extremely sensitive electrochemical sensor of ascorbic acid based on a mixture of NiAl_2_O_4_-NiO nanoparticles that, produced in a single step using the sol–gel method, on an ITO electrode. This new sensing platform is useful for the detection of ascorbic acid with a wide range of concentrations extending from the attomolar to the molar. SEM micrographs show the porous structure of the NiAl_2_O_4_-NiO sample, with a high specific surface area, which is beneficial for the catalytic performance of the nanocomposite. An XRD diffractogram confirmed the existence of two phases, NiAl_2_O_4_ and NiO, both corresponding to the face-centred cubic crystal structure. The performances of the modified electrode, as a biomolecule, in the detection of ascorbic acid was evaluated electrochemically by cyclic voltammetry and chronoamperometry. The sensor exhibited a sensitive electrocatalytic response at a working potential of *E* = +0.3 V vs. Ag/Ag Cl, reaching a steady-state current within 30 s after each addition of ascorbic acid solution with a wide dynamic range of concentrations extending from attolevels (10^−18^ M) to molar (10 mM) and limits of detection and quantification of 1.2 × 10^−18^ M and 3.96 × 10^−18^ M, respectively. This detection device was tested for the quantification of ascorbic acid in a 500 mg vitamin C commercialized tablet that was not pre-treated.

## 1. Introduction

The design of an electrochemical (bio)sensor for ex vivo detection of ascorbic acid (AA) has gained significant interest due to AA’s crucial role in various physiological processes, including the immune system, iron absorption, cholesterol, protein metabolism [1], and collagen synthesis [2]. Indeed, ascorbic acid (AA), commonly called vitamin C, is a well-known antioxidant added to preserve the appearance of foods and drinks and to prevent unwanted changes in color, flavor, and browning [3,4]. The European Food Safety Authority suggests a daily intake of around 90 mg for healthy adults; exceeding this amount can provoke kidney and digestive problems. Conversely, a deficiency in AA causes scurvy, a pathology related to collagen synthesis [5,6]. Therefore, the design of a sensor capable of detecting and analyzing both low and high concentrations of this target analyte (AA) will be a significant asset in the field of biosensing, and this constitutes one of the objectives of this study.

In biosensing, an electrode is a key component used as a solid support for the immobilization of biomolecules (enzymes, antibodies, and nucleic acid) and the movement of electrons. Furthermore, with advancements in nanotechnology, the design and selection of nanoparticles for the surface functionalization of an electrode are crucial, as they possess unique optical, electrical, thermal, and catalytic properties that can significantly influence sensing performance. These nanomaterials can be metallic nanoparticles [7], carbon nanotubes [8], or nanoparticles made of semiconductor materials [9]. The latter have dimensions comparable to the dimensions of biomolecules (enzymes, antigens/antibodies, DNA, etc.), making increased contact surface possible. Nanoparticles can be synthesized with different methods, such as sol–gel synthesis, the co-precipitation method [10], microwave heating [11], chemical synthesis [12], or the hydrothermal method [13]. The sol–gel technique produces a small particle size, high surface area, good conductivity, and high stability, which makes this technique suitable for nanoparticles synthesis.

However, despite a high demand and the intense research activity devoted to improving the performance of biosensors, few commercial achievements have seen the light. The major difficulty that slows the industrial development of biosensors is linked to a reduction in biosensor activity when biomolecules are attached to the surface of the sensor.

In response to these challenges, ongoing research focuses on the development of a novel sensor that eradicates the need to immobilize biological molecules on the surface of the electrode. This strategy enhances the preservation of the biological activity of the target, thereby protecting it from eventual reaction with molecules at the surface, providing time saving, cost-effectiveness, stability, and longevity.

Nickel oxide (NiO) nanoparticles have generated significant interest in sensing research, particularly in the development of biomolecule-sensing electrodes. These nanoparticles have distinct optical, electronic, and physiochemical properties, making them ideal for a variety of applications [14]. Indeed, the use of NiO nanoparticles in sensing electrodes has the potential to significantly improve the sensitivity and reliability of biomolecules detection, contributing to advances in biomedical and environmental monitoring applications [15]. For example, Chen et al. investigated the use of NiO nanoparticles for DNA adsorption [16]. They selected NiO from six metal oxides (NiO, CoO, ZnO, TiO_2_, CeO_2_, and Fe_3_O_4_) to extract DNA from a complex matrix (serum) and noticed that NiO was the best promising candidate for extracting DNA from biological samples. The adsorption of DNA was explained by the fact that nucleic acids have a negatively charged phosphate backbone that interacts strongly with metal oxides.

On the other hand, nickel aluminate (NiAl_2_O_4_) is a spinel-type material with superior catalytic properties, and is used in various products such as supercapacitors, batteries, electrocatalysts, etc. As far as we know, there are few reports regarding the inclusion of cubic NiAl_2_O_4_ nanoparticles in biomolecules sensors. In fact, we found only two publications in this scope: the first reports the use of these nanoparticles for the detection of gallic acid in food samples [17], while the second reports 5-hydroxymethyl furfural detection in coffee [18].

This study represents a contribution to this field, as we propose a nanocomposite of NiO and NiAl_2_O_4_ nanoparticles as a new sensing platform. This mixture of NiAl_2_O_4_-NiO, prepared in one step using an easy sol–gel method, was deposited on an ITO electrode.

To the best of the authors’ knowledge, this proposed platform has never been used for the design of electrochemical biosensors. The suggested approach combines the advantage of the catalytic properties of NiAl_2_O_4_ nanoparticles [17,18] with the high reactivity of NiO nanostructures [16]. The choice of ascorbic acid as the biological analyte for detection stemmed from the fact that it is the most common small biological molecule found in human blood and can effectively penetrate the pores within our designed nanostructures. Furthermore, this proposed detection strategy eliminated the requirement to immobilize biological molecules on the electrode surface to detect target analytes. This resulted in advantages such as reduced analysis time, improved cost-effectiveness, enhanced sensor stability, and extended sensor lifetime.

The suggested detection procedure was tested for the quantification of ascorbic acid in a real sample of a 500 mg vitamin C commercialized tablet.

## 2. Results

### 2.1. Synthesis of NiAl_2_O_4_-NiO Nanostructures

We synthesized NiAl_2_O_4_-NiO nanostructures using the sol–gel method. First, we prepared a mixture containing 4.246 g of aluminum, 1.645 g of nickel nitrate, and 5.812 g of starch. This mixture was then added to 100 mL of ethanol. The solution was magnetically stirred at 100 °C for 7 h, resulting in the formation of a gel. The gel was inserted into a furnace where combustion occurred and a dark brown powder was produced. The powder was then calcined at a temperature of ≈ 800 °C in air for 4 h. For better fixation of nanostructures on the substrate, the powder was mixed with nafion polymer at a concentration of 5% and drop-casted on an ITO substrate. Nafion, due to its film-forming properties, enabled better adherence of NiAl_2_O_4_-NiO nanostructures to the ITO surface electrode.

### 2.2. Morphological and Structural Characterization

#### 2.2.1. SEM Analysis

**Figure 1:**
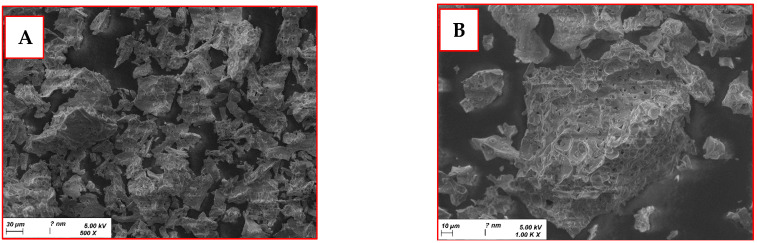

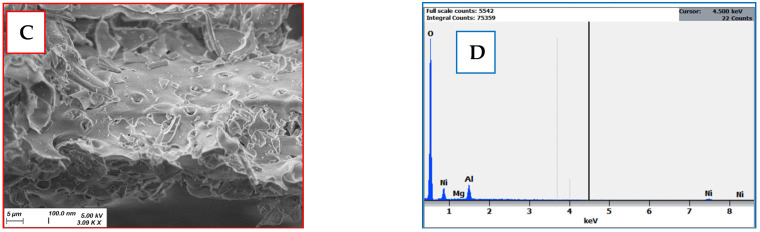
Scanning electron microscopy images of NiAl_2_O_4_-NiO nanocomposites at different magnifications of (**A**) ×500, (**B**) ×1000, and (**C**) ×3000; and (**D**) SEM–EDS elemental analysis.

NiAl_2_O_4_-NiO nanocomposites were characterized by SEM. Images presented in Figure 1 show SEM micrographs of the nanocomposite at different magnifications with energy dispersive X-ray spectroscopy SEM–EDS elemental analysis. These nanocomposites had a rough and porous surface with an agglomeration of nanometric particles. The interconnected porous structures can be attributed to the release of volatile gases during the combustion phase [19]. This high degree of porosity led to a large specific surface area, enhancing the catalytic performance of the nanoparticles.

#### 2.2.2. XRD Analysis

**Figure 2:**
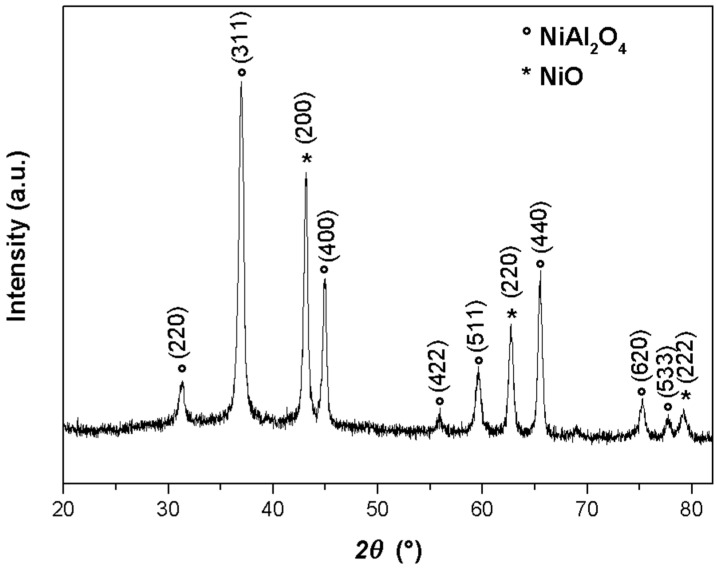
X-ray diffractogram of NiAl_2_O_4_-NiO nanocomposites.

The X-ray diffraction of the NiAl_2_O_4_-NiO nanocomposite given in Figure 2 shows the existence of two phases. The main diffraction peaks at 2θ of 31.38°, 37.02°, 45.02°, 49.30°, 55.94°, 59.66°, 65.54°, 74.54°, and 77.70° were assigned to the cubic spinal structure of NiAl_2_O_4_ with the space group Fd3m [20]. The intense and fine diffraction peaks indicate that the structure of these aluminate spinels had a high degree of long-range order. The peaks appearing at 2θ = 43.16°, 62.74°, and 79.38° were relative to the NiO crystal planes, and corresponded to the face-centred cubic crystal structure of NiO [21].

#### 2.2.3. FT-IR Analysis

**Figure 3:**
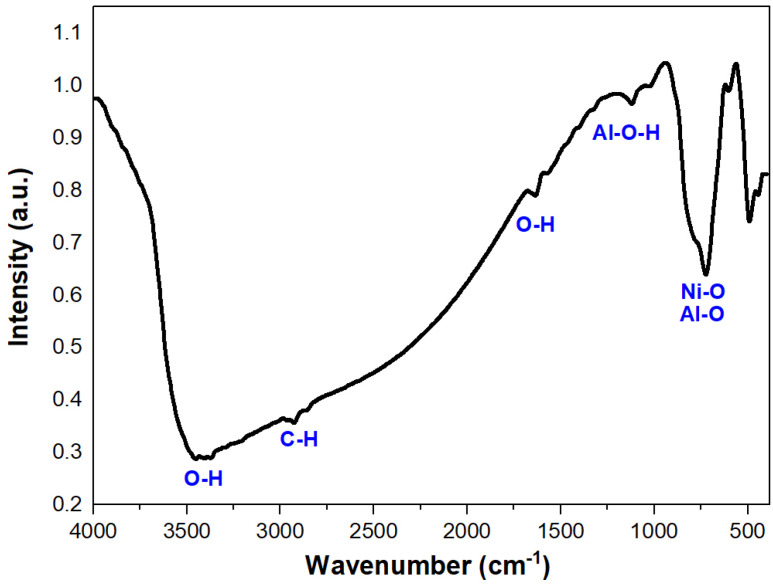
FT-IR spectrum of NiAl_2_O_4_-NiO nanocomposites.

FT-IR spectroscopy allowed an examination of the structural features of the NiAl_2_O_4_-NiO nanocomposite. As presented in Figure 3, small absorption bands were observed at about 3460, 2918, 2860, 1635, 1120, and 1130 cm^−1^. Bands at 3460 and 1635 cm^−1^ can be attributed to the antisymmetric/symmetric stretching and bending vibrational modes of the hydroxyl group, respectively [22]. This could be the result of moisture from the air seeping into samples during KBr pellets preparation. Small peaks at about 2918 and 2860 cm^−1^ can be attributed to C–H stretching vibrational modes [23]. Bands at 1126 and 1058 cm^−1^ were due to the symmetrical bending vibration of the Al–O–H group [24]. The presence of four prominent vibrational bands observed at around 720, 600, 490, and 440 cm^−1^ suggested the formation of the NiAl_2_O_4_ aluminate spinel structure. The lowest mode is attributed to the stretching vibration of the metal–oxygen at the octahedral site, whereas the first three modes are assigned to the intrinsic stretching vibrations of the metal–oxygen at the tetrahedral sites [22]. 

#### 2.2.4. Electrochemical Characterization

**Figure 4:**
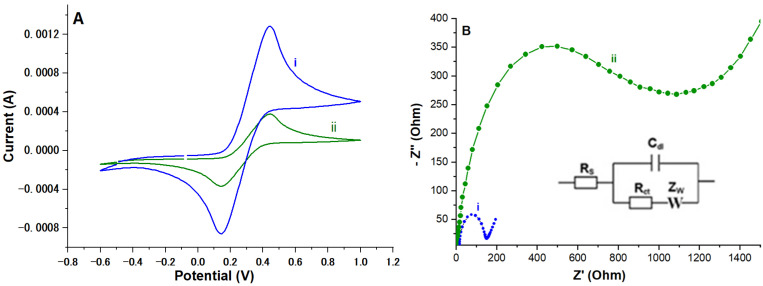
(**A**) Voltammetric curves at a scanning speed of 0.1 Vs^−1^ and (**B**) EIS profiles recorded for a frequency range from 0.1 Hz to 100 kHz on an ITO electrode (i) before and (ii) after modification by NiAl_2_O_4_-NiO nanocomposites in a 5 mM [Fe(CN)_6_]^3−^/^4−^ solution, pH = 7.4. Ag/AgCl was the reference electrode. Inset is the Randles equivalent circuit model used for EIS measurements, where R_s_ is the electrolyte resistance, R_ct_ is the charge transfer resistance, C_dl_ is the double layer capacitance, and W is the Warburg diffusion impedance.

The use of a redox probe in solution generally makes it possible to monitor changes at an electrode surface [25]. In aqueous electrochemistry, a K_3_[Fe(CN)_6_]/K_4_[Fe(CN)_6_] probe is often used because the Fe(III)/Fe(II) couple provides access to various information relating to the functionalization of the surface of the electrode. Cyclic voltammetry (CV) curves recorded for the redox couple K_3_[Fe(CN)_6_]/K_4_[Fe(CN)_6_] on the bare ITO working electrode and on the same electrode after functionalization with NiAl_2_O_4_-NiO are presented in Figure 4A. The blue curve (curve (i)) shows the reversible process of a redox reaction of the Fe (III)/Fe (II) couple at the surface of the bare ITO electrode (before modification). After modification by NiAl_2_O_4_-NiO, the peaks of the redox process substantially decreased compared to the bare electrode (Figure 4A(ii)). This shows that the current measured on the NiAl_2_O_4_-NiO nanostructures reduced the faradic process, and consequently the conductivity of the ITO electrode, confirming the formation of a film completely adsorbed at the surface of the electrode [25].

This was confirmed by electrochemical impedance spectroscopy (Figure 4B), considered one of the most powerful surface characterization techniques. In Nyquist plots, these spectra exhibit a characteristic shape, well recognized in various types of uncoated electrodes or thin dielectric coatings with leaks: a slightly compressed semicircle which can be represented by a parallel combination of a resistor (representing coating resistance and/or electrochemical reaction) and a capacitor (representing coating capacitance and/or an electrical double layer), along with a low-frequency linear segment, describable by Warburg impedance depicting a diffusion-controlled electrochemical reaction [25]. Assuming that the semicircle’s diameter in the Nyquist plot reflected charge–transfer resistance, a roughly 6-fold increase in this value was observed after functionalization of the bare ITO electrode (Figure 4B curve (i)) with NiAl_2_O_4_-NiO nanostructures (Figure 4B curve (ii)). This led to a significant decrease in the conductivity between the electrode and the water-soluble redox active probe, and confirmed the subsequent immobilization of NiAl_2_O_4_-NiO nanocomposites on the surface of the ITO electrode (Figure 4B).

### 2.3. Electrochemical Performance of NiAl_2_O_4_-NiO/ITO Electrode

**Figure 5:**
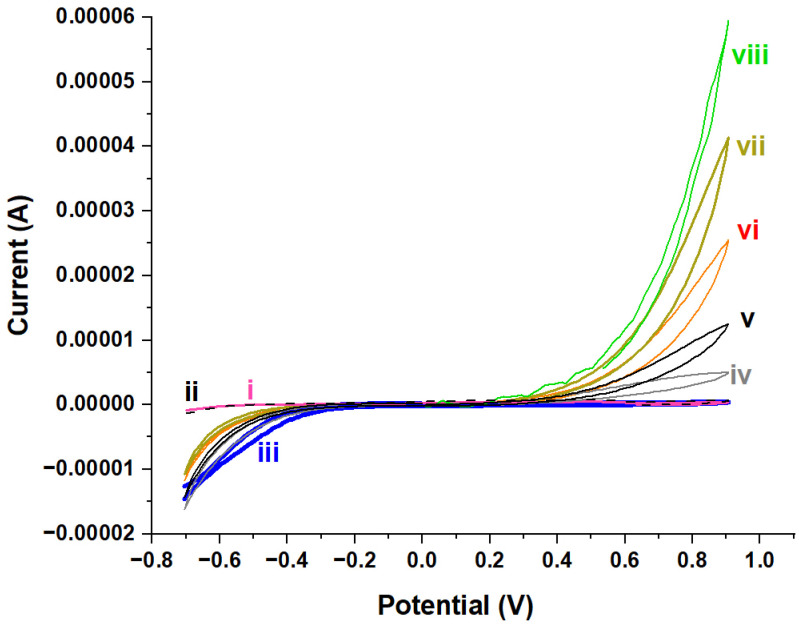
Voltammetric curves obtained by cyclic voltammetry of the bare ITO electrode (i) before and (ii) after addition of 1 nM of AA, and of the ITO electrode modified by NiAl_2_O_4_-NiO nanostructures (iii) before and after addition of (iv) 0.1 nM, (v) 0.2 nM, (vi) 0.3 nM, (vii) 0.4 nM, and (viii) 0.5 nM of ascorbic acid. Scan speed = 0.05 Vs^−1^, in a phosphate buffer with pH = 7.4 vs. a Ag/AgCl reference electrode.

The performance of the modified electrode for the detection of ascorbic acid as a biomolecule were evaluated by cyclic voltammetry and chronoamperometry. Figure 5 presents the CV curves of an ITO electrode modified by NiAl_2_O_4_-NiO nanocomposites in a solution containing a phosphate buffer at pH = 7.4 (Figure 5(iii)). 

The addition of 0.1, 0.2, 0.3, 0.4, and 0.5 nM of ascorbic acid generated a remarkable increase in the oxidation current of the NiAl_2_O_4_-NiO. This effect was not observed for the bare ITO electrode, even at 1 nM of ascorbic acid (Figure 5(ii)). This enhancement in the anodic signal is attributed to the catalytic activity of NiAl_2_O_4_-NiO nanostructures towards ascorbic acid oxidation. Indeed, this catalytic effect, ensured by the NiAl_2_O_4_-NiO nanocomposite, can be explained by its semiconductor properties, which in the presence of AA considerably increased the conductivity of the film and accelerated the electronic transfer towards the surface of the ITO electrode. Interestingly, the catalytic effect seems to have been strictly dependent on the presence of ascorbic acid with the NiAl₂O₄-NiO nanostructures. This behavior was not observed with the Fe (III)/Fe (II) redox couple, in which, instead, we encountered a blocking effect explained by the formation of a film of NiAl₂O₄-NiO nanostructures at the ITO surface.

**Figure 6:**
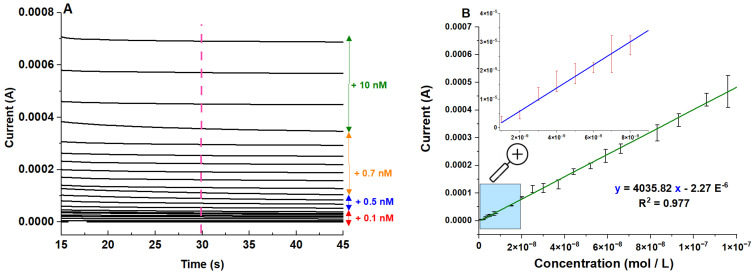
(**A**) Chronoamperograms of the NiAl_2_O_4_-NiO/ITO electrode following the addition of ascorbic acid, a potential of E = +0.3 V (arrows indicate amounts of added ascorbic acid). (**B**) Corresponding calibration curve of NiAl_2_O_4_-NiO-modified ITO electrode according to AA concentrations ranging from 0.1 nM to 10 µM. Measurements were performed at t = 30 s in a phosphate buffer with pH = 7.4 vs. a Ag/AgCl reference electrode.

Furthermore, chronoamperometric responses of the modified electrodes following successive additions of AA solution were evaluated (Figure 6).

To assess both reproducibility and repeatability at each concentration of the AA solution, six runs were performed. Three runs were performed using the same electrode (testing reproducibility), while the other three runs were performed with different electrodes (testing repeatability). As shown in Figure 6A, the sensor showed a sensitive and rapid response, reaching a steady state after approximately 30 s (Figure 6A). The current was an affine function of the AA concentration for concentrations ranging from 0.1 nM to 10 µM with a correlation coefficient equal to R^2^ = 0.978 (Figure 6B).

Due to the wide dynamic range of the sensor, we determined the lower (limit of detection) and upper (saturation of the electrode) limits separately by performing separate experiments in the same conditions, as indicated in Figure 6. This allowed us to determine a detection limit of 1.2 × 10^−18^ M and a saturation limit that was recorded at concentrations of 10 mM. The limits of detection and quantification were, respectively, 1.2 × 10^−18^ M and 3.96 × 10^−18^ M, calculated according to 3 times s/m and 10 times s/m criteria, respectively, where ‘’s’’ was the standard deviation of the peak current of low concentration of the analyte and ‘‘m’’ was the slope of the related calibration curve (five runs).

**Figure 7:**
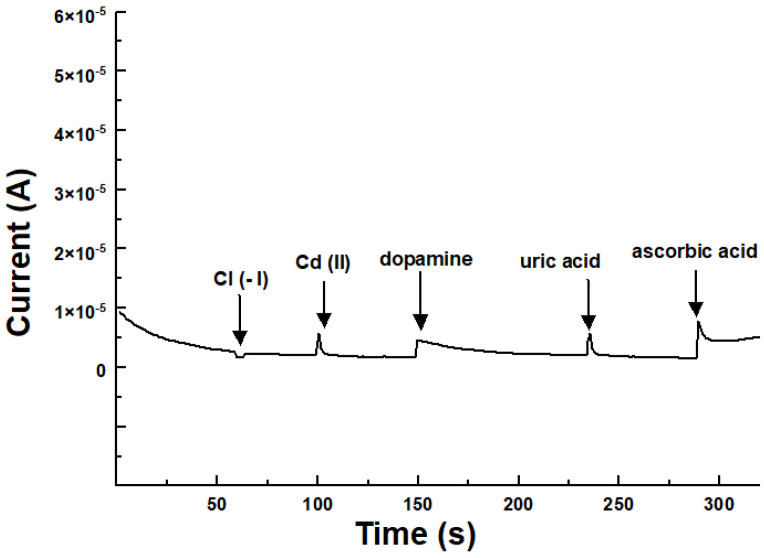
Current–time response of NiAl_2_O_4_-NiO/ITO electrode at a potential of E = +0.3 V vs. Ag/AgCl in a phosphate buffer for additions of 1 µM Cl (-I), 1 µM Cd (II), 1 µM uric acid, 1 µM dopamine, and 0.1 nM of ascorbic acid.

Moreover, the proposed sensor was selective towards AA (Figure 7). In fact, a major problem encountered in the determination of AA is interference from uric acid and dopamine. As shown in Figure 7, one can easily conclude that this sensor is selective to AA, even with significant excesses of uric acid and dopamine. 

This sensor was then tested in a real matrix using a commercially available tablet of 500 mg of vitamin C that was not pre-treated. Results were satisfactory, perfectly matching with previous results. It is noteworthy that the biosensor we developed maintained excellent sensitivity and stability for the detection of ascorbic acid for several months.

## 3. Discussion

These results are promising, as they show the unique properties of the elaborated electrode compared to those of electrodes available on the market. Indeed, our electrode presents spectacular catalytic activity in the presence of ascorbic acid, making it an innovative electrochemical biosensor with a wide dynamic range and a low detection limit (Table 1).

NiAl_2_O_4_-NiO on an ITO electrode can be used to detect ascorbic acid at low or high concentrations. The proposed method is inexpensive, as we did not use a biological molecule in the electrode preparation, which can lead to electrode degradation or instability and contribute to the high cost of the electrode. In the future, we plan to use this electrode to detect other types of molecules, such as DNA, which is an anionic acid that may have molecular properties similar to those of ascorbic acid.

As a limitation of our study, we observed certain variability in the current response of the suggested sensor for the same concentrations (Figure 6 B), indicating that the sensor might not yet meet the stringent requirements necessary for clinical applications demanding high accuracy and precision. To address these issues, we are currently conducting additional experiments to further refine the sensor. These include optimizing the proportion of NiAl_2_O_4_-NiO, adjusting the concentration of nafion, and exploring different pH levels. Our goal is to ensure the sensor’s robustness and reliability in clinical settings.

## 4. Materials and Methods

Nickel (II) nitrate hexahydrate (Ni(NO_3_)_2_·6H_2_O), aluminum nitrate nonahydrate (Al(NO_3_)_2_·9H_2_O), sodium hydrogen phosphate (Na_2_HPO_4_), sodium dihydrogen phosphate (NaH_2_PO_4_), K_3_[Fe(CN)_6_]/K_4_[Fe(CN)_6_], nafion, ascorbic acid, and potassium chloride (KCl) were purchased from Merck Tunisia (https://bestunisie.com/ (accessed on 13 June 2024)) and used as received without further purification for all experiments. We used deionized water for aqueous solutions preparation (Milli-Q Millipore system, Tunis, Tunisia), the final resistivity being at least 18 MΩ·cm^−1^. Electrochemical analysis was carried out using a three-electrode electrochemical cell with an Ag/AgCl (sat.KCl) reference electrode (Metrohm Tunisie: https://www.interchimie.tn/ (accessed on 13 June 2024)), platinum wire (Pt, Metrohm, Metrohm Tunisia: https://www.interchimie.tn (accessed on 13 June 2024)) as an auxiliary electrode, and ITO substrates as the working electrode. These ITO electrodes have a geometric area of 1 cm^2^. NiAl_2_O_4_-NiO nanostructures covered the whole surface of the submerged portion of the ITO electrode. All electrochemical measurements were taken at room temperature in 0.1 M phosphate buffered solution (PBS) at pH = 7.4. EIS measurements in this study were carried out using a 5 mM K_3_[Fe(CN)_6_]/K_4_[Fe(CN)_6_] redox solution and the frequency was swept from 100 kHz to 0.1 Hz with an amplitude modulation of ± 20 mV. Stock solutions of ascorbic acid (c = 1 mmol/L) and uric acid (c = 1 mmol/L) were prepared in deionized water, while the stock dopamine solution (c = 1 mmol/L) was prepared in 10^−3^ mol/L of HCl solution to avoid dopamine oxidation by the dissolved oxygen in aqueous solution. Phosphate buffers were prepared in deionized water according to the manufacturer’s recommendations by dissolving PBS tablets, and the pH was adjusted by adding aqueous hydrochloric solution.

A SEM (JEOL, LV-6380, Sollentuna, Sweden) equipped with an energy-dispersive X-ray spectroscope (EDS) for elemental analysis was employed to analyse morphology. An automated Bruker D8 advance X-ray diffractometer with Cu Kα (*λ* = 1.54 Å) in 2θ ranging from 20 to 70° was used to determine structural characteristics. FT-IR spectroscopy was conducted on a Bruker Vertex 80/80v spectrometer (Bruker Optics Inc., Billerica, MA, USA) with KBr pellets in transmission mode within the range of 400–4000 cm^−1^. Electrochemical measurements (cyclic voltammetry (CV), and chronoamperometry (CA)) were carried out on an Autolab PC-controlled PGstat M204 equipped with a FRA32 impedance module using NOVA^®^1.11 software. A conventional three-electrode glass cell was used for all electrochemical experiments. For electrochemical impedance spectroscopy (EIS) measurements, this study used the hexacyanoferrate system K_3_[Fe(CN)_6_]/K_4_[Fe(CN)_6_] over a frequency range that extended from 100 kHz to 100 mHz with an amplitude of ± 10 mV.

## 5. Conclusions

In this study, we developed a simple, selective, and extremely sensitive method for high detection of biological molecules, particularly ascorbic acid, in the attomolar range. The process of preparing this biosensor was performed in a single step, which consisted of modifying an ITO electrode with NiAl_2_O_4_-NiO nanocomposite, produced by the sol–gel method. Our electrochemical sensor revealed a remarkably wide response range, extending from attomolar concentrations to molar concentrations, attributed to the catalytic activity of ascorbic acid towards NiAl_2_O_4_-NiO. These results are promising, as they show the unique sensing properties of our electrode.

## 6. Patents

This work has been submitted for a national patent under this number: TN 2022/0331.

## Figures and Tables

**Table 1:** Comparison of the present study with other published studies of ascorbic acid detection.

Electrode Material	Linear Range	Detection Limit	Ref.
gC_3_N_4_/MWNTs/GO ^1^	0.2 to 7.5 mM	96 μM	Wang et al. (2022) [26]
p-Purpald@GCE ^2^	33.2 to 76.9 µM	392 nM	Varatharajan et al. (2024) [27]
n-MgF/SPE/EμPAD ^3^	0 to 80 μM	2.44 μM	Gautam et al. (2024) [28]
Fe_3_O_4_/GCE ^4^	1050 to 2300 µM	95 µM	Gaya et al. (2024) [29]
NiAl_2_O_4_-NiO/ITO	3.96 × 10^−9^ nM to 10 mM	1.2 × 10^−9^ nM	Present work

^1^ MWNTs: multi-walled carbon nanotubes, **^2^** Purpald: 4-amino-3-hydrazino-5-mercapto-1,2,4-triazole, **^3^** EμPAD: microfluidic paper-based device, SPE: screen-printed electrode, ^4^ GCE: glassy carbon electrode.

## Data Availability

The original contributions presented in the study are included in the article, further inquiries can be directed to the corresponding author.
